# Optimizing Granulocyte-Colony Stimulating Factor Dosing Scheme in Healthy Stem Cell Donors for Allogenic Transplantation: A Retrospective Cohort Study of Single Dose vs. Split Dose

**DOI:** 10.7759/cureus.76553

**Published:** 2024-12-29

**Authors:** Sidika Gülkan Özkan, Ali Kimiaei, Seyedehtina Safaei, Meral Sönmezoğlu, Gulderen Yanikkaya Demirel, Sema Aktas, Sinem Özdemir, Helin Serda Özalp, Hasan Atilla Özkan

**Affiliations:** 1 Hematology, Bahçeşehir University, Istanbul, TUR; 2 Infectious Diseases, Yeditepe University Hospital, Istanbul, TUR; 3 Immunology, Yeditepe University, Istanbul, TUR; 4 Therapeutic Apheresis, Yeditepe University Hospital, Istanbul, TUR; 5 Adult Hematology and Bone Marrow Transplantation, Medical Park Göztepe Hospital, Istanbul, TUR; 6 Coordinator, Turkish Stem Cell Coordination Center, Republic of Turkey Ministry of Health, Ankara, TUR

**Keywords:** allogeneic stem cell transplantation, apheresis, cd34⁺, filgrastim, g-csf, granulocyte colony-stimulating factor, stem cell mobilization

## Abstract

Objectives

The optimal dosing schedule strategy for granulocyte colony-stimulating factor (G-CSF) in healthy stem cell donors remains controversial. This study aimed to compare the efficacy of once-daily versus twice-daily G-CSF administration in allogeneic stem cell donors.

Materials and methods

We retrospectively analyzed data from 388 healthy unrelated donors (282 males, 106 females) who underwent stem cell mobilization at our center between September 2018 and June 2022. Donors received daily either a single dose of 10 μg/kg/day or two doses of 5 μg/kg/day of filgrastim.

Results

The split-dose group had significantly higher CD34+ cell counts in peripheral blood (p<0.001), total collected CD34+ cell counts (p<0.001), and total collected CD34+ cell counts per donor body weight (p<0.001) than the single-dose group with one apheresis procedure on the fifth day of GCSF. Multivariable analysis revealed that split-dose G-CSF administration was independently associated with higher peripheral blood CD34+ cell count (p<0.001) and higher total collected CD34+ cell count per donor body weight (p<0.001). Coincidentally, the split-dose group also had higher rates of catheter use (p = 0.003).

Conclusion

Split-dose G-CSF administration led to better stem cell mobilization than once-daily dosing. These findings contribute to optimizing G-CSF regimens for allogeneic stem cell donation, potentially improving transplantation outcomes.

## Introduction

Allogeneic hematopoietic stem cell transplantation (allo-HSCT) has become a crucial curative treatment for numerous malignant and nonmalignant hematological disorders. Bone marrow (BM), peripheral blood (PB), and cord blood unit (CBU) can be used as stem cell sources in allo-HSCT. Since the mid-1990s, the use of granulocyte colony-stimulating factor (G-CSF) to mobilize peripheral blood stem cells has become the globally preferred method for harvesting from healthy donors [1]. Due to faster engraftment, improved graft-versus-leukemia effect, and absence of donor exposure to general anesthesia and bone marrow harvesting, peripheral blood (PB) has become the preferred stem cell source for allo-HSCT in recent years [2].

Effective mobilization is a crucial step for successful allo-HSCT. The success of stem cell harvesting depends on various interrelated factors, including the dosage and administration schedule of G-CSF, the use of central venous catheters, and donor characteristics such as gender, age, body mass index (BMI), and ethnicity [3-6].

The choice of recombinant human G-CSF (rhG-CSF) for stem cell mobilization typically involves either lenograstim or filgrastim [1]. Although various studies report different doses of G-CSF for mobilization [7-9], guidelines generally recommend a total G-CSF dose of 10 µg/kg, administered either once daily or in divided doses. This regimen is preferred because a 10 µg/kg dose over five consecutive days achieves a higher CD34+ blood peak and greater CD34+ cell yield compared to lower doses [1]. However, it has been observed that administering doses exceeding 10 μg/kg is associated with an increased incidence of side effects [10].

The effect of a once-daily versus twice-daily G-CSF dosing schedule on CD34+ cell yield or blood counts was assessed in many studies with variable results. While some studies suggest that twice-daily dosing may be more effective in increasing CD34+ cell yield, there is also evidence indicating that once-daily dosing can be equally effective in achieving adequate CD34+ cell counts [11-15].

The main aim of this study is to evaluate the effect of the G-CSF administration scheme with one apheresis procedure on pre-apheresis peripheral CD34/uL, product CD34/uL, and product CD34/donor kg. We aim to add valuable information to the ongoing debate on optimizing G-CSF dosing schemes for stem cell mobilization in allogeneic transplantation settings.

## Materials and methods

This study adhered to the principles outlined in the Declaration of Helsinki and received approval from the local Institutional Ethics Committee on October 4, 2023 (approval number 2023-17/02). All donors provided written informed consent before participation.

Study design and participants

We retrospectively analyzed data from unrelated donors who volunteered for allogeneic stem cell transplantation at our center between September 2018 and June 2022. All donors were unrelated to their intended recipients and were recruited through the TÜRKÖK program. These donors are participants of the study published in *Transfusion *with the title “Prediction of CD34+ hematopoietic stem cells in healthy allogeneic stem cell donors on the Optia cell separator based on CE2: Which formula is more correlated with actual CD34+?” [16]. Of the 397 donors in the published study, 388 (282 males and 106 females) donors for whom the G-CSF application scheme was available were included in the current study.

G-CSF administration and stem cell collection

Donors were divided into two groups based on their filgrastim (G-CSF) administration regimen without any randomization: (1) A single daily dose of 10 micrograms/kg/day and (2) Two doses of 5 micrograms/kg/day (split-dose)

The following data were collected and compared between two groups: hematological parameters before and on the fifth day after filgrastim administration, including the donor's complete blood count, peripheral blood CD34+ percentage, and absolute values before apheresis were recorded. Donor weight, total blood volume of the donor, donor's processed blood volume, procedure time, amount of product obtained (ml), and number of leukocytes in the product were recorded. The percentage of product CD34+ with the first apheresis and its absolute value was also recorded on the apheresis form. As the number of apheresis procedures is variable depending on the body weight of the recipient, it was not evaluated in our study. All patient and donor information was extracted retrospectively from the hospital's electronic medical records. CD34 counts were conducted following International Society of Hematotherapy and Graft Engineering (ISHAGE) guidelines, utilizing a Navios flow cytometry system and StemKit CD34 kit (Beckman Coulter, Brea, USA) [17].

Hematopoietic stem cell collection was performed using a Spectra Optia apheresis device (Terumo, Tokyo, Japan). The collection flow rate was determined by the device's program after inputting complete blood count values. The anticoagulant ratio ranged from 12:1 to 14:1.

Statistical analysis

IBM SPSS Statistics for Windows, Version 25.0 (IBM Corp., Armonk, USA) was used for statistical analysis. Histograms and Q-Q plots were used to examine the conformity of the variables to a normal distribution. Descriptive statistics were presented using mean ± standard deviation for continuous variables due to normality of distribution and frequency (percentage) for categorical variables. Continuous variables were analyzed with the Student’s t-test due to normality of distribution, and categorical variables were analyzed with the chi-square test. Multivariable linear regression analysis was used to determine independently associated factors with the outcomes. Factors were analyzed with the univariable linear regression analysis, and then statistically significant factors were analyzed with the multivariable linear regression analysis. Two-tailed p-values of less than 0.05 were considered statistically significant. According to descriptive statistics (effect size = 0.495) in the study by Yano et al. [18], a sample size of 87 for each group (174 in total) achieves 90% power at the two-sided 0.05 significance level. The sample size was calculated using PASS 11 software (NCSS, Kaysville, USA) and two-sample t-test power analysis [19].

## Results

We included 388 donors (282 males and 106 females) in the study, mean age was 32.08 ± 7.97 (range 19 - 51) years. 218 (56.19%) donors had received single-dose G-CSF and 170 (43.81%) donors had received split-dose G-CSF. The catheter use percentage (p=0.003) was significantly higher in the split-dose group than in the single-dose group. We found no significant differences between G-CSF administration groups in terms of age, sex, and body weight.

**Figure 1:**
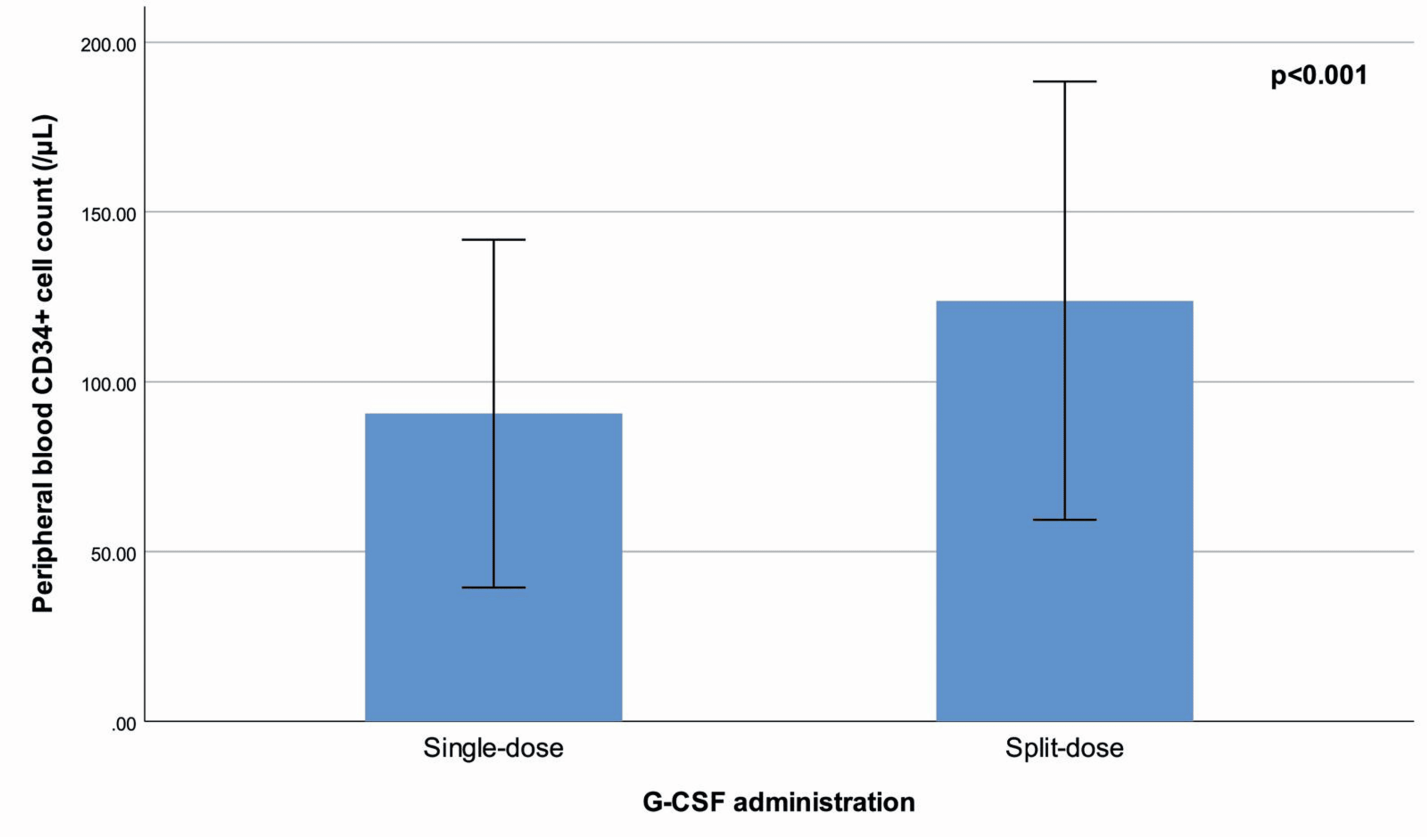
Peripheral blood CD34+ cell count (/μL) (mean ± standard deviation) with regard to G-CSF administration. Peripheral blood CD34+ cell count (/μL) (mean ± standard deviation) with regard to granulocyte-colony stimulating factor (G-CSF) administration. The figure compares the peripheral blood CD34+ cell counts between donors who received a single daily dose of G-CSF (10 μg/kg/day) and those who received a split dose (5 μg/kg twice daily). The split-dose group demonstrated significantly higher peripheral blood CD34+ cell counts compared to the single-dose group, with a p-value < 0.001, indicating a statistically significant difference between the two groups.

**Figure 2:**
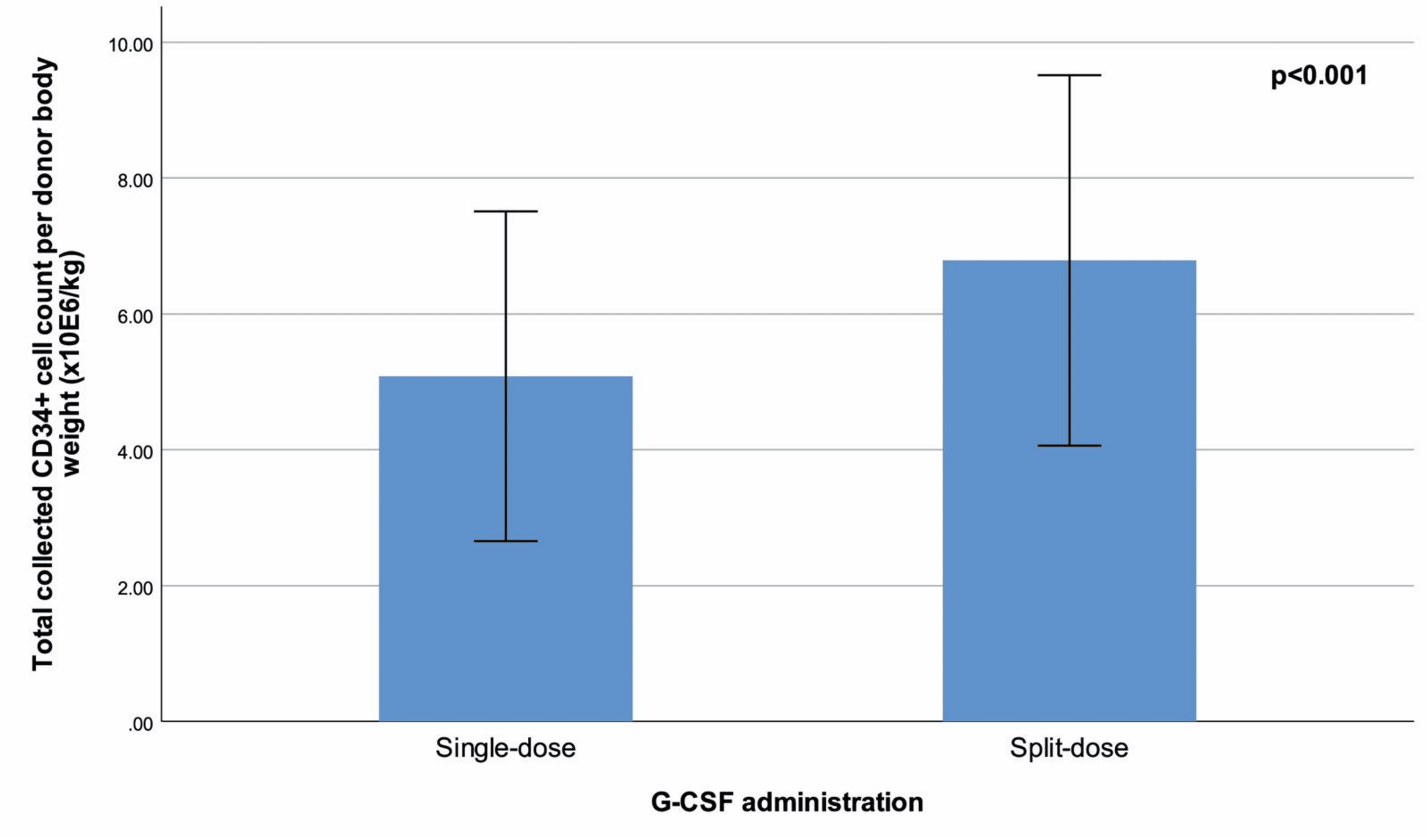
Total collected CD34+ cell count per donor body weight (x10E6/kg) (mean ± standard deviation) with regard to G-CSF administration. Total collected CD34+ cell count per donor body weight (x10^6/kg) (mean ± standard deviation) with regard to granulocyte-colony stimulating factor (G-CSF) administration. The figure compares the total CD34+ cells collected per donor body weight between donors who received a single daily dose of G-CSF (10 μg/kg/day) and those who received a split dose (5 μg/kg twice daily). The split-dose group exhibited significantly higher CD34+ cell counts per donor body weight compared to the single-dose group, with a p-value < 0.001, indicating a statistically significant difference between the two groups.

Peripheral blood CD34+ cell count (p<0.001) (Figure 1), peripheral blood WBC (p<0.001), total collected CD34+ cell count (p<0.001), total collected CD34+ cell count per donor body weight (p<0.001) (Figure 2), product volume (p<0.001), duration of process (p<0.001), and processed blood volume (p=0.001) were significantly higher in the split-dose group than in the single-dose group. There were no significant differences between G-CSF administration groups in terms of total blood volume, product CD34+ cell count, product hematocrit, and product WBC (Table 1).

**Table 1:** Summary of variables with regard to granulocyte-colony stimulating factor (G-CSF) administration. Descriptive statistics were presented using mean ± standard deviation for continuous variables and frequency (percentage) for categorical variables. † Student's t test, # Chi-square test.

Variables	Total (n=388)	Single-dose (n=218)	Split-dose (n=170)	p
Age (years)	32.08 ± 7.97	32.11 ± 8.02	32.04 ± 7.94	0.923^†^
Sex				
Male	282 (72.68%)	165 (75.69%)	117 (68.82%)	0.132^#^
Female	106 (27.32%)	53 (24.31%)	53 (31.18%)	
Body weight (kg)	79.03 ± 15.26	80.28 ± 14.55	77.44 ± 16.03	0.069^†^
Catheter use				
No	338 (87.11%)	200 (91.74%)	138 (81.18%)	0.003^#^
Yes	50 (12.89%)	18 (8.26%)	32 (18.82%)	
Total blood volume (mL)	4975.30 ± 855.42	5029.33 ± 778.93	4906.02 ± 942.34	0.169^†^
Peripheral blood CD34+ cell count (/μL)	105.18 ± 59.67	90.62 ± 51.18	123.86 ± 64.55	<0.001^†^
Peripheral blood WBC (x10E3/μL)	44.35 ± 14.82	38.42 ± 12.80	51.95 ± 13.76	<0.001^†^
Product CD34+ cell count (/μL)	1477.08 ± 614.27	1494.75 ± 611.96	1454.42 ± 618.28	0.522^†^
Total collected CD34+ cell count (x10E6)	453.36 ± 208.02	405.06 ± 197.75	515.29 ± 204.96	<0.001^†^
Total collected CD34+ cell count per donor body weight (x10E6/kg)	5.83 ± 2.70	5.08 ± 2.43	6.79 ± 2.73	<0.001^†^
Product volume (mL)	314.00 ± 91.09	273.25 ± 79.82	366.25 ± 77.01	<0.001^†^
Product hematocrit (%)	1.84 ± 0.65	1.80 ± 0.58	1.88 ± 0.74	0.217^†^
Duration of process	231.23 ± 54.11	219.59 ± 55.98	246.16 ± 47.78	<0.001^†^
Product WBC (x10E3)	204.79 ± 40.44	203.87 ± 40.09	205.96 ± 40.97	0.613^†^
Processed blood volume (L)	9.15 ± 2.39	8.80 ± 2.51	9.59 ± 2.16	0.001^†^

According to multivariable linear regression analysis results, high body weight (p = 0.037) and split-dose G-CSF administration (p<0.001) were independently associated with the higher peripheral blood CD34+ cell count (Table 2).

**Table 2:** Association between factors and peripheral blood CD34+ cell count (/μL), linear regression analysis results. CI: Confidence interval; G-CSF: Granulocyte-Colony Stimulating Factor

Variables	Univariable			Multivariable		
	Unstandardized coefficients (95% CI)	Standardized coefficients	p	Unstandardized coefficients (95% CI)	Standardized coefficients	p
Age	-0.706 (-1.451 - 0.040)	-0.094	0.064			
Sex, Male	14.828 (1.527 - 28.129)	0.111	0.029	10.752 (-3.579 - 25.083)	0.080	0.141
Body weight (kg)	0.487 (0.099 - 0.875)	0.125	0.014	0.448 (0.028 - 0.867)	0.114	0.037
G-CSF administration, Split-dose	33.240 (21.689 - 44.790)	0.277	<0.001	35.247 (23.785 - 46.709)	0.293	<0.001
Adjusted R^2^	-			0.097		
Regression model	-			F=14.918, p<0.001		

According to multivariable linear regression analysis results, the male sex (p = 0.002) was independently associated with the higher product CD34+ cell count, and the high duration of the process (p = 0.049) was independently associated with the lower product CD34+ cell count (Table 3).

**Table 3:** Association between factors and product CD34+ cell count (/μL), linear regression analysis results. CI: Confidence interval; G-CSF: Granulocyte-Colony Stimulating Factor

Variables	Univariable			Multivariable		
	Unstandardized coefficients (95% CI)	Standardized coefficients	p	Unstandardized coefficients (95% CI)	Standardized coefficients	p
Age	-4.732 (-12.428 - 2.964)	-0.061	0.227	-	-	-
Sex, Male	317.823 (183.770 - 451.876)	0.231	<0.001	258.988 (94.286 - 423.690)	0.188	0.002
Body weight (kg)	7.187 (3.224 - 11.150)	0.179	<0.001	3.169 (-1.406 - 7.745)	0.079	0.174
G-CSF administration, Split-dose	-40.335 (-164.004 - 83.335)	-0.033	0.522	-	-	-
Catheter use, Yes	-12.581 (-195.810 - 170.647)	-0.007	0.893	-	-	-
Duration of process	-3.305 (-4.392 - -2.218)	-0.291	<0.001	-2.245 (-4.489 - -0.001)	-0.198	0.049
Processed blood volume (L)	-42.679 (-68.025 - -17.333)	-0.166	0.001	-18.935 (-71.052 - 33.181)	-0.074	0.475
Adjusted R^2^	-			0.118		
Regression model	-			F=13.969, p<0.001		

According to multivariable linear regression analysis results, split-dose G-CSF administration (p<0.001), catheter use (p = 0.040), and high processed blood volume (p = 0.029) were independently associated with the higher total collected CD34+ cell count per donor body weight. In addition, high age (p=0.001) was independently associated with the lower total collected CD34+ cell count per donor body weight (Table 4).

**Table 4:** Association between factors and total collected CD34+ cell count per donor body weight (x10E6/kg), linear regression analysis results. CI: Confidence interval; G-CSF: Granulocyte-Colony Stimulating Factor

Variables	Univariable			Multivariable		
	Unstandardized coefficients (95% CI)	Standardized coefficients	p	Unstandardized coefficients (95% CI)	Standardized coefficients	p
Age	-0.053 (-0.087 - -0.020)	-0.157	0.002	-0.055 (-0.086 - -0.023)	-0.161	0.001
Sex, Male	0.213 (-0.391 - 0.818)	0.035	0.488			
G-CSF administration, Split-dose	1.706 (1.190 - 2.222)	0.314	<0.001	1.560 (1.032 - 2.087)	0.287	<0.001
Catheter use, Yes	1.125 (0.328 - 1.921)	0.140	0.006	0.818 (0.039 - 1.596)	0.102	0.040
Duration of process	0.007 (0.002 - 0.011)	0.131	0.010	-0.004 (-0.012 - 0.004)	-0.075	0.351
Processed blood volume (L)	0.152 (0.041 - 0.264)	0.135	0.008	0.198 (0.020 - 0.375)	0.175	0.029
Adjusted R^2^	-			0.133		
Regression model	-			F=12.824, p<0.001		

## Discussion

In this study, we compared the effects of once-daily versus twice-daily administration of filgrastim on stem cell mobilization in healthy stem cell donors with the aim of determining the optimal dosing strategy for maximizing CD34+ cell yield. Our findings demonstrated that the split-dose regimen resulted in significantly higher peripheral blood CD34+ cell counts, total collected CD34+ cell counts, and total collected CD34+ cell count per donor body weight compared to the single-dose regimen with one apheresis procedure. Since this study had the highest number of donors in the literature to date with a two-dose administration scheme of 5 micrograms/kg/day (administered twice daily) and a single daily dose of 10 micrograms/kg/day [20], our study adds valuable information to the ongoing debate on optimizing G-CSF application regimens for stem cell mobilization in allogeneic transplantation settings.

Current guidelines from the American Society for Blood and Marrow Transplantation (ASBMT) endorse a single daily dose of 10 μg/kg/day of filgrastim for mobilizing peripheral blood progenitor cells (PBPCs) in healthy donors, with leukapheresis typically beginning on the fifth day [1]. Although split dosing has been reported to increase cell yield, single dosing is recommended because split dosing is less convenient for donors [1]. This regimen is widely adopted due to its balance between efficacy and safety. However, evidence from our study and others suggests that twice-daily dosing may offer superior stem cell mobilization, potentially improving the success rates of allogeneic transplantation. The challenge lies in balancing these benefits against the increased risk of adverse effects, such as bone pain, headaches, and procedural complexities associated with split dosing [18].

Our results align with several studies that have reported improved outcomes with split dosing. Arbona et al. [21] and Ponisch et al. [22] both found higher cell yields with twice-daily application, although they used different total daily G-CSF doses. Ponisch et al. [22] specifically found that administering G-CSF every 12 hours at doses of 6 or 8 μg/kg resulted in superior CD34+ cell yields compared to a once-daily dose of 10 μg/kg. Additionally, a prospective trial in healthy donors showed that twice-daily injections (2 × 5 μg/kg) resulted in a significantly higher CD34+ cell harvest than a single application (1 × 10 μg/kg) [12]. Similarly, Lee et al. [23] observed that split-dose regimens yielded higher CD34+ cell counts in pediatric patients, further supporting the potential advantages of more frequent dosing in certain donor populations. This suggests that the frequency of dosing may play a critical role in enhancing stem cell mobilization, possibly due to more consistent stimulation of the bone marrow.

However, the literature is not unanimous on this point. Some studies have found no significant differences between once-daily and twice-daily dosing regimens. For instance, a retrospective study by Pinar et al. [15] found no differences in CD34+ cell yields between donors who received once-daily or twice-daily G-CSF dosing. Similarly, Komeno et al. [13] compared G-CSF 400 μg/m² once daily and 200 μg/m² twice daily, resulting in similar CD34+ cell yields. Additionally, Anderlini et al. [11] found no significant difference in CD34+ cell yields between once-daily and twice-daily G-CSF administration. This discrepancy could be attributed to differences in study design, like the target value of CD34, the small number of participants (power of the study), population characteristics, and the specific G-CSF dosing protocols used (2 × 6 µg/kg and 1 × 12 µg/kg). We believe that it is not appropriate to evaluate the efficacy of G-CSF administered to the donor with calculations based on the weight of the recipient.

The pharmacological profile of G-CSF provides a compelling rationale for the potential superiority of split-dose regimens. Evidence suggests that a once daily G-CSF application might be suboptimal due to its pharmacokinetic properties. In both healthy donors and cancer patients, G-CSF reaches its maximum serum concentration within 2-8 hours after subcutaneous administration [24]. Moreover, G-CSF (filgrastim) has a relatively short elimination half-life of approximately 3-4 hours, regardless of the route of administration [21,22]. This pharmacokinetic profile indicates that a single daily dose may not maintain optimal G-CSF levels throughout the 24-hour period. Consequently, several studies in healthy donors have suggested that splitting the G-CSF dosage into two injections with a 12-hour interval might lead to a higher CD34+ cell harvest [21,22]. Our findings align with this pharmacological rationale, demonstrating improved outcomes with twice-daily administration. This pharmacokinetic perspective not only supports our results but also provides a mechanistic explanation for the potential benefits of split-dose regimens in stem cell mobilization.

In a large study following thousands of healthy donors for over 10 years on average, filgrastim was not associated with increased incidence of myeloid or lymphoid malignancies, other cancer types, autoimmune diseases, or thrombotic events [25]. While significant differences in adverse effects between once-daily and split-dose G-CSF regimens have not been consistently observed, some research has reported increased toxicities with higher G-CSF doses. These include bone pain, fatigue, headaches, and chills, which can impact donor comfort and willingness to participate in future donations [12,26-28]. Chen et al. [10] found an increased frequency of bone pain, headaches, and chills with G-CSF doses exceeding 10 μg/kg/day. However, Beelen et al. [29] compared 10 μg/kg/day once daily to 8 μg/kg/day split into two doses and found similar frequencies of adverse events in both groups. Our study prioritized efficacy evaluation and did not analyze side effect details. Future prospective comparative studies should evaluate both efficacy and side effects concurrently [25]. Short-term safety concerns remain paramount when considering modifications to established G-CSF dosing protocols.

Additionally, the correlation between donor characteristics and mobilization efficiency is a critical factor in optimizing G-CSF dosing. Our multivariable analysis indicated that factors such as high body weight, male sex, and younger age were independently associated with higher CD34+ cell yields. A retrospective study of 86 patients by Cetin et al. [30] found that in individuals with BMI > 25 kg/m², once-daily dosing resulted in a higher CD34+ cell yield. This finding is further supported by a large study using the Center for International Blood and Marrow Transplant Research (CIBMTR) database, which evaluated the impact of donor BMI on G-CSF-mobilized peripheral blood stem cell (PBSC) yield in 20,884 healthy unrelated donors [31]. They found that obese and severely obese donors had higher stem cell collection yields than those with normal or overweight BMI [31]. Increasing the G-CSF dose improved yields in normal and overweight donors, but for obese and severely obese donors, doses beyond 780 µg and 900 µg per day, respectively, did not increase yield [31]. This suggests a maximum effective G-CSF dose for PBSC mobilization in obese and severely obese donors, beyond which higher doses of G-CSF add no increased yield [31]. Thus, personalized dosing strategies based on donor characteristics might be beneficial.

This study has several limitations that should be acknowledged. First, we were unable to access data on the side effects experienced by donors during G-CSF administration, limiting our ability to fully assess the safety profile of the dosing regimens. Additionally, we did not record which biosimilars were used in the study. Another limitation is the anonymity maintained by the TÜRKÖK program, where the follow-up of donors and recipients is conducted at different centers, preventing us from linking donors to recipients and evaluating transplantation outcomes such as engraftment time and long-term survival. Lastly, the retrospective, single-center design may introduce selection bias and limit the generalizability of the findings to other populations. Additionally, we did not evaluate other factors such as donor preferences, cost considerations, and practical logistics, which could influence the feasibility and acceptability of different dosing regimens. These factors suggest the need for prospective, multi-center studies that include comprehensive side effect monitoring and long-term follow-up to validate our findings and guide clinical practice.

## Conclusions

In conclusion, our study contributes to the growing body of evidence suggesting that twice-daily administration of G-CSF may lead to more efficient stem cell mobilization in healthy donors. However, given the variability in results across different studies and the potential for increased side effects with higher doses, a one-size-fits-all approach may not be optimal, and donor-specific factors should be carefully considered when determining the most appropriate G-CSF dosing regimen. Future research should focus on larger, prospective trials that consider donor characteristics and long-term outcomes to definitively establish the most effective and safe G-CSF administration protocol for healthy stem cell donors.
